# A revised radiocarbon chronology for the mammoth bone structures and associated features at Mezhyrich, Ukraine

**DOI:** 10.12688/openreseurope.20112.1

**Published:** 2025-07-25

**Authors:** Wei Chu, Pavlo Shydlovskyi, Andreas Maier

**Affiliations:** 1 World Archaeology, Universiteit Leiden, Leiden, South Holland, 2333CC, The Netherlands; 2 Department of Archaeology and Museum Studies, Taras Shevchenko National University of Kyiv, Kyiv, 01033, Ukraine; 3 Institute of Prehistoric Archaeology, University of Cologne, Cologne, North Rhine-Westphalia, 50969, Germany

**Keywords:** Late Upper Palaeolithic, Mezhyrich, Mammoth Bone Structures, radiocarbon dating, isotopic analysis, human-environment interactions, settlement chronology

## Abstract

Mammoth Bone Structures are a distinct archaeological phenomenon typically ascribed to the Late Pleistocene in East-Central Europe though their chronology and use remain debated. By reviewing the history of research and excavation at the Mezhyrich site, this article presents new ^14^C results from Mezhyrich (Cherkasy Oblast, Ukraine), revising the chronology of an Upper Palaeolithic Mammoth Bone Structure. Using meso-mammal remains from cultural layers, the results provide a more constrained timeline than previous models based on mammoth bones. The findings date Mammoth Bone Structure 4 and its context to c. 18,248–17,764 years cal BP with a site duration lasting between 0–429 years. This shorter chronology is consistent with a single occupation model but cannot exclude repeated occupation within maximally a few centuries at around 18 ka cal BP contributing to the hypothesis that the structure served as a dwelling, offering insights into Late Upper Palaeolithic life patterns in Eastern Europe.

## Plain Language Abstract

Here we present new radiocarbon dates for a mammoth bone structure at Mezhyrich, Ukraine, refining its timeline. By dating small mammal remains instead of mammoth bones, we establish a more precise age of around 18,248–17,764 years ago. The structure appears to have been used for up to 429 years, supporting the idea that it served as a dwelling. These findings help us better understand how people lived in Eastern Europe during the Late Upper Paleolithic.

## Introduction

Open-air Upper Palaeolithic sites in East-Central Europe offer valuable insights into human activity and biogeography during the Late Pleniglacial, a period of intense environmental change ( Verpoorte, 2009). Many of these sites, characterised by deep loessic sedimentary sequences, contain abundant lithic, faunal, and osseous assemblages with embedded environmental proxies to help determine contemporaneous environmental factors ( Chu & Nett, 2021). Notably in this region, some of these sites are found in direct association with mammoth bone accumulations posited to be among the earliest evidence of built structures that exhibit spatial and seasonal organisation ( Iakovleva & Djindjian, 2005; Iakovleva *et al.*, 2012; Shipman, 2015; Shydlovskyi *et al.*, 2022).

Many of these constructions have been previously interpreted as the remains of domestic structures ( Aurenche *et al.*, 2013; Gladkih *et al.*, 1984; Iakovleva, 2021; Iakovleva & Djindjian, 2018; Klein, 1974; Pidoplichko & Allsworth-Jones, 1998; Soffer, 1985; Soffer *et al.*, 1997). However, since most were excavated decades ago, the question has reemerged as to whether they functioned primarily as dwellings or if they represent sites of other functions, such as bone beds, food caches, burials, religious traditions, or ritualised middens that may have served as early monuments ( Djindjian, 2015; Gavrilov, 2024; Graeber & Wengrow, 2021, p. 73; Pryor *et al.*, 2020; Sablin *et al.*, 2023; Zheltova, 2024).

**Figure 1.**
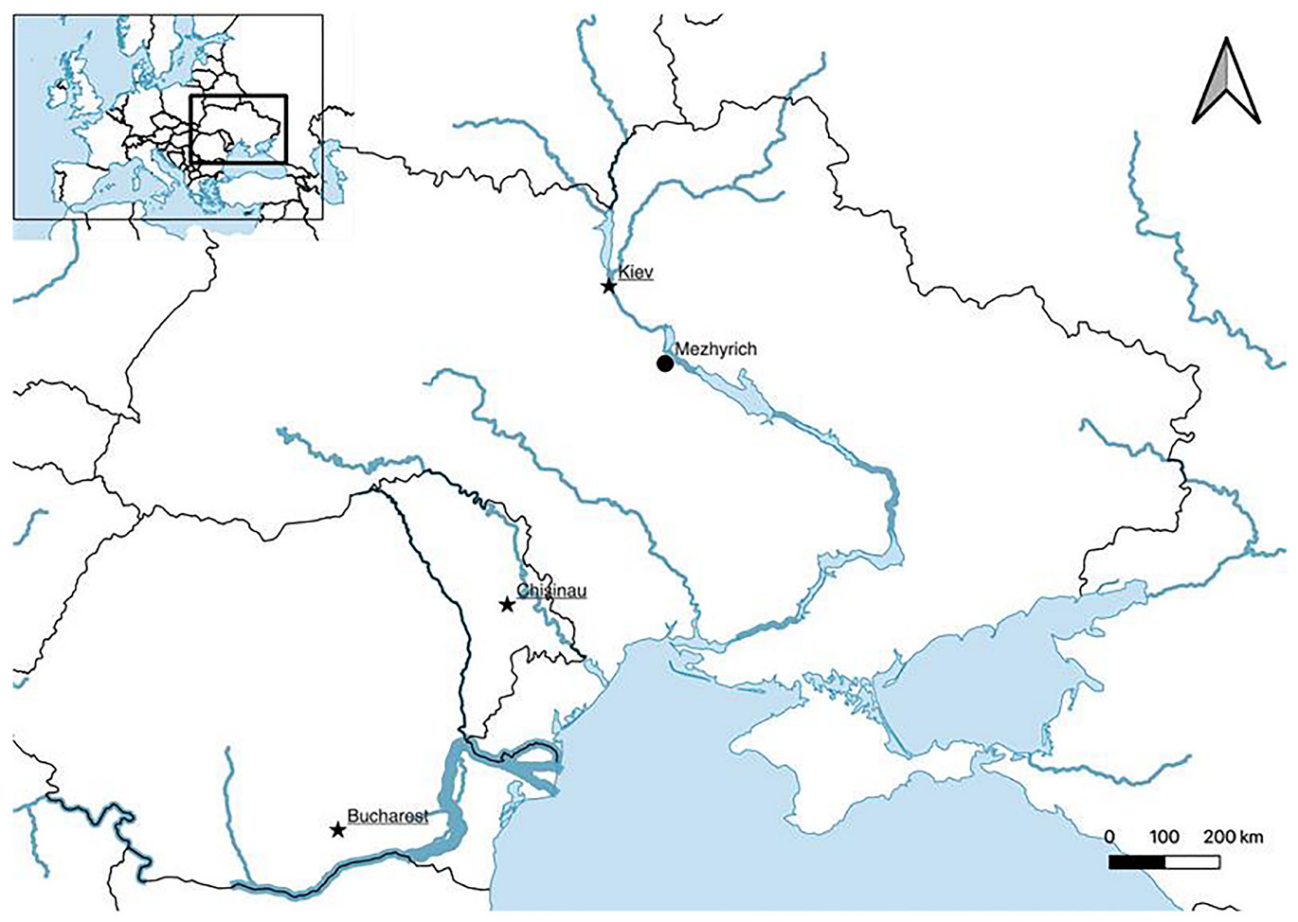
Map of Mezhyrich's location within East Central Europe.

A key site to this debate is Mezhyrich (Межиріч), situated in the Middle Dnieper Basin of Ukraine known for its exceptional preservation of four Mammoth Bone Structures (MBSs) between 12–24 m ^2 ^in diameter ( Figure 1). These structures are associated with peripheral features and artefacts including artefact-filled pits, hunting weapons, ivory, and bone ornaments, as well as delimited activity areas with butchered animal remains and workshops with dense cultural layers, each delimited into “economical settlement units” with the MBS as the focal point (e.g. Units 1–4; Shydlovskyi *et al.*, 2019).

Still, two key challenges hinder thorough interpretations of Mezhyrich:

1. There is still a coarse understanding of the temporal scale of use of MBSs ( Gaudzinski-Windheuser, 2011; Iakovleva *et al.*, 2012; Shydlovskyi *et al.*, 2023). While the Mezhyrich MBSs were repeatedly used, radiometric ages for the specific occupation durations are scarce. Previous ages for Mezhyrich’s MBS 4 have largely been based on disparate radiocarbon methods using mammoth remains that have likely been primarily obtained through scavenging. As a result, ages may not coincide with the cultural deposition and may contribute to overestimates of the age of the anthropogenic activities at the site ( van der Plicht & Palstra, 2016). It is still unclear how often Mezhyrich MBS 4 was occupied and for how long these occupations lasted.
2. There is still a lack of reliable ages to establish correlations between the cultural layers found within MBS 4 and associated peripheral features, such as artefact dense areas (e.g., *Toptalishche* areas) and nearby pits (e.g., Pit 6). Sediment accumulation within MBS 4 may be independent of external loess deposition and more closely related to the frequency, duration, and mode of human use.

**Figure 2.**
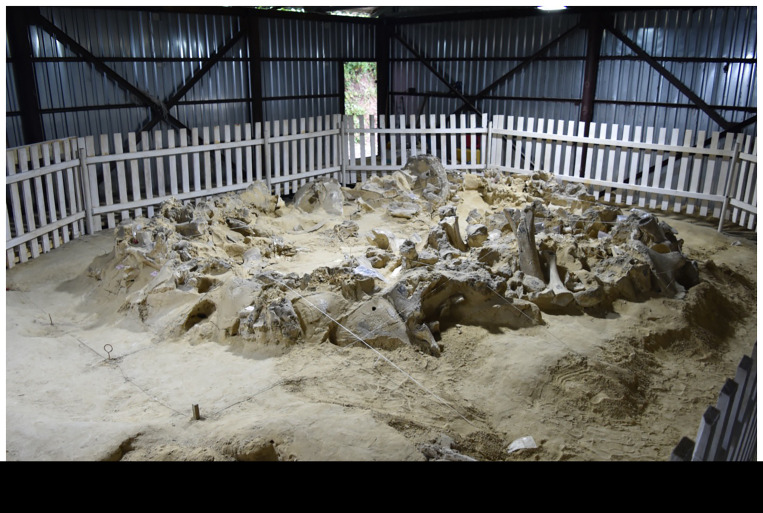
Photograph of Dwelling 4 during excavation.

To address these research gaps, this study presents new AMS radiocarbon ages and isotopic data obtained from medium-sized mammal remains directly recovered from archaeological layers within Mezhyrich. This study focuses on the dating and analysis of MBS 4 (Unit 4), the only one of these structures that contains well-provenienced artefacts within cultural layers from which to obtain direct ages, as well as Pit 6 (Unit 2) and the external saturated cultural layer ( *Toptalishche*; Unit 1; Figure 2). The primary objective is to evaluate the occupation history of MBS 4 and its implications for the broader settlement history of the site during the Late Pleistocene.

Based on this refined chronology, we contribute to the interpretation of MBS 4 as a multi-phasic dwelling structure and propose that such structures played significant roles in the East Central European life patterns during the Late Upper Palaeolithic. This adds to a better understanding of the function and chronology the MBS, providing insights into the past human behaviours and adaptations in this region.

## Background

In 1930, M.Ia. Rudynskyi’s excavation at the Mizyn (Mezin) settlement uncovered a cluster he interpreted as a “small residential building” with “curtain walls” ( Rudynskyi, 1954, pp. 35–37). Around the same time, Yefimenko emphasised studying Upper Palaeolithic residential structures to understand the social relationships and worldview of prehistoric communities hypothesising dwellings at Eastern European sites like Kostenki I and II, Hintsi (Gontsy), and Suponievo ( Yefimenko, 1953).

The study of Palaeolithic dwellings subsequently became a focus of Eastern European archaeology, aided by large-area excavation techniques that enabled recording various settlement features simultaneously ( Shovkoplias, 1965, p. 260). Significant contributions came from the Desna expedition at sites near Novhorod-Siverskyi, Chulatovo, and Pushkari where at Pushkari I, Boriskovskii proposed a residential structure reconstruction ( Boriskovskii, 1940).

Further excavations at the Pushkari Cape at sites like Pushkari I, Pogon (aka Pushkari IX, layer 3 and Pushkari VIII), and Bugorok (aka Pushkari IX), revealed substantial evidence of residential structures ( Voevodskii, 1950). At the Chulatovo II settlement (1936–1938), Voevodskyi uncovered remains of a small structure, delimited by vertically placed mammoth tusks and long bones ( Voevodskii, 1952, pp. 105–106).

**Figure 3.**
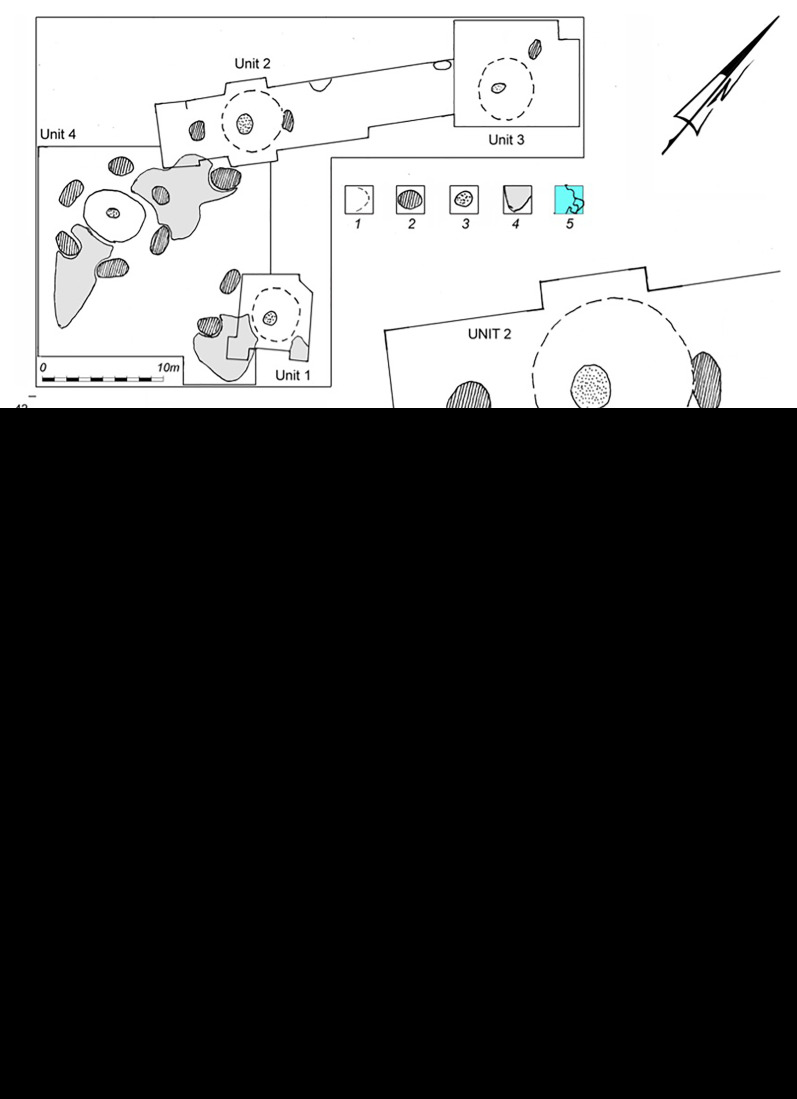
Plan of Mezhyrich site: 1 – limits of mammoth bone structures; 2 – pits; 3 – hearths; 4 – limits of dense anthropogenic layers (“Toptalishche”); 5 – plots from which samples were obtained.

Initial excavations at Mezhyrich (1966–1974), yielded Palaeolithic artefacts and three discrete MBSs, each c. 6 m in diameter ( Pidoplichko, 1976). A lack of detailed provenience recording led to a significant loss of archaeological material and information concerning cultural layers and spatial arrangements ( Gladkikh & Kornietz, 1979b). The subsequent discovery of a fourth structure ( *Dwelling 4;* MBS 4) in 1976 has since exposed a complex of surrounding pits and activity areas that exhibit diverse artefacts embedded within distinct cultural layers ( Figure 3; Gladkih *et al.*, 1984; Soffer *et al.*, 1997).

**Figure 4.**
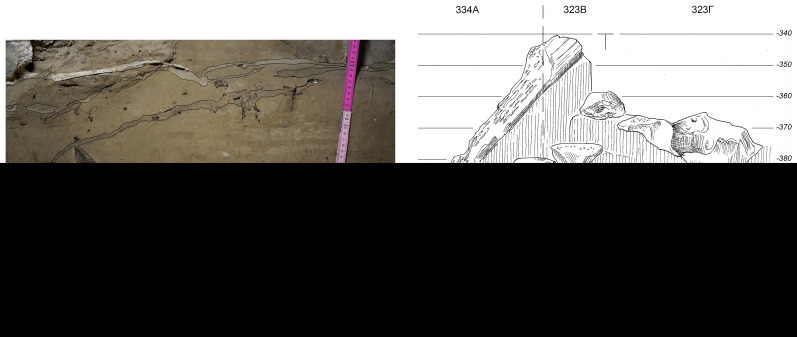
Site stratigraphy of MBS4 and with three archaeological layers (after Tsvirkun *et al.*, 2021).

MBS 4 provides evidence of past domestic activities through the accumulation of at least three internal cultural layers and the presence of butchered remains of juvenile and adolescent mammoths within the surrounding pits ( Tsvirkun *et al.*, 2021; Figure 4). The mammoth bones used in the construction of MBS 4 were sourced from a minimum of 37 individuals, predominantly from natural accumulations, though some were obtained in anatomical position as evidenced by at least one freshly deceased mammoth carcass ( Gladkih *et al.*, 1984).

Subsequent studies have provided environmental context for the Mezhyrich site ( Table 1) notably demonstrating a local dynamic environment that may have been warmer and more humid than other contemporary periglacial areas of Europe. Long-term trends in stable isotope analyses revealed that the initially strongly diversified herbivore niches of the mammoth steppe collapsed during the Last Glacial Maximum ( Reiss *et al.*, 2023) in Central Europe. While early post-LGM isotope analysis on faunal remains from Barmaky (19 ka; north-western Ukraine) still show a strongly diversified niche spectrum for herbivores ( Reiss *et al.*, In Review), measurements on mammoth remains from younger specimens in Eastern Europe give mixed signals, but may point to a disappearance of their optimal habitat and increasing competition with other large herbivores, particularly at the southern fringes of their distribution in the East European Plain ( Drucker *et al.*, 2018). The associated eponymous Epigravettian technological tradition, known as the Mezhyrich industry, is believed to have abruptly ceased around c. 13 ka ago, coinciding with the local disappearance of mammoths ( Gladkikh, 1977; Nuzhnyi, 2008; Shydlovskyi *et al.*, 2020).

**Table 1.** Previous contextual studies on the Mezhyrich site.

Study	Findings	Reference
Stable isotopes	• Low ^15^N due to collapse of ecological niche c. 18–17 ka and focus on mature grasses. • changes in regional flora and climatic conditions may have altered the food resources available to mammoths, potentially leading to direct competition with horse populations.	( Drucker *et al.*, 2014; Drucker *et al.*, 2018)
Microstratigraphy	• The activities of Epigravettian hunter-gatherers between 18.3–17.4 ka took place during a cold period characterised by strong climatic and environmental contrasts.	( Haesaerts *et al.*, 2015)
Anthracology	• The major charcoal signal is preserved within the microscopic part of the archaeological sediments underlying the intensity of taphonomic processes. • Birch and willow were located along the riverbanks.	( Marquer *et al.*, 2012)
Micromammals	• Environment was cold but not as dry as in a typical periglacial area, warmer and wet, a cold mesophilic forest-steppe.	( Rekovets *et al.*, 2014)
Mammals	• The results exhibit a usage of the pit as a dump area of food and technical remains from mainly mammoth, hare, and fox processing, and bone fuels. • The information about mammoth procurement by the last Palaeolithic hunter–gatherers in Eastern Europe allows to document the hunting activities on the mammoth populations, which were probably already weakened at the end of the Pleistocene.	( Péan, 2015)
Palynology	• Plant cover during Upper Palaeolithic had a mosaic structure.	( Komar, 2015)
Malacology	• The molluscan fauna from the *Toptalishche* is characteristic of drier and milder local conditions. • The molluscan fauna from below the *Toptalishche* indicates stadial loessic deposits.	( Prisiazhniuk, 2015)

### Stratigraphy

Initial geomorphological data suggested a Late Pleistocene chronology for the site dating the cultural layers to the Late Glacial period no earlier than 17–16 ka BP during cold and arid climatic conditions, though less severe than those during the Last Glacial Maximum (LGM; Kornietz *et al.*, 1981; Velichko *et al.*, 1993a; Velichko *et al.*, 1993b; Velichko *et al.*, 1994. During excavations south of MBS 4 in 1978, two distinct cultural layers separated by a sterile layer (10–20 cm thick) were identified; the lower layer was linked to the construction phase of the MBS, while the upper layer was associated with the settlement’s final occupation phase. These two discrete cultural layers were interpreted as episodic and intermittent occupations of the site by prehistoric groups, indicative of transient hunting camps ( Gladkikh & Kornietz, 1979a, pp. 11–13).

Further geoarchaeological research has since corroborated the multi-layered stratigraphy of the site with microstratigraphic analyses of the lithostratigraphy, around the excavation perimeter, targeting the anthropogenic layers of the 1 ^st^, 2 ^nd^, and 4 ^th^ MBSs. This research revealed three cultural layers intercalated by sterile layers, thereby elucidating the spatial and temporal relationships among different Units at the site ( Haesaerts *et al.*, 2015; Shydlovskyi *et al.*, 2019).

### Chronology

34 previously reported radiocarbon ages from Mezhyrich have suggested an occupation of the site between c. 24–14 ka calibrated Before Present (cal BP; Table 2). However, these ages vary in ^14^C-measurement methodologies, sample materials, and selection methods resulting in a wide distribution ( Gladkikh & Kornietz, 1979b; Gladkikh & Kornietz, 1982; Haesaerts, 2006; Haesaerts *et al.*, 2015; Shydlovskyi *et al.*, 2023; Soffer, 1985; Soffer, 1993).

**Table 2.** Previous radiocarbon ages from Mezhyrich.

Unit, context	Method	Dated material *taxa*	Lab-ID	^14^C Age (yrBP)	± 1- sigma	Calibrated age (95.4% probability)	Source
DW 4, cultural layer	Conventional	Burned bone ( *Mammuthus*)	KI-1054	17850	950	24110–19484	( Gladkikh & Kornietz, 1979b, p. 16)
Cultural layer (1978)	Conventional	Burned tooth ( *Mammuthus*)	KI-1055	18020	600	23278–20408	( Gladkikh & Kornietz, 1979b, p. 16)
Cultural layer (1976)	Conventional	Burned bone ( *Mammuthus*)	KI-1056	18470	550	23741–21050	( Gladkikh & Kornietz, 1979b, p. 16)
DW 1, cultural layer (1966)	Conventional	Burned bone ( *Mammuthus*)	KI-1057	19100	500	24263–22050	( Gladkikh & Kornietz, 1979b, p. 16)
DW 1, cultural layer (1966)	Conventional	Bone ( *Mammuthus*)	KI-1058	19280	600	24893–22090	( Gladkikh & Kornietz, 1979b, p. 16)
DW 4, cultural layer	Conventional	Burned bone ( *Mammuthus*)	QC-900 В	15245	1080	21743–15980	( Gladkikh & Kornietz, 1982, p. 18)
DW 1, cultural layer	Conventional	Burned bone ( *Mammuthus*)	QC-897	14320	270	18211–16771	( Soffer, 1985, p. 143)
DW 3, cultural layer	Conventional	Tooth ( *Mammuthus*)	GIN-2593	14700	500	19056–16611	( Soffer, 1985, p. 143)
DW 2, cultural layer	Conventional	Burned bone ( *Mammuthus*)	GIN-2595	14530	300	18619–16982	( Soffer, 1985, p. 143)
DW 4, cultural layer	Conventional	Burned bone ( *Mammuthus*)	GIN-2596	14300	300	18221–16641	( Soffer, 1985, p. 143)
DW 1	AMS	Tooth (collagen) ( *Mammuthus*)	OxA-709	12900	200	16070–14591	( Soffer, 1985, p. 26)
DW 2	AMS	Tooth (collagen) ( *Mammuthus*)	OxA-712	14400	250	18225–16988	( Soffer, 1985, p. 26)
DW 3	AMS	Tooth ( *Mammuthus*)	AA-1317	14420	190	18138–17097	( Soffer, 1985, p. 26)
DW 1	AMS	Femur ( *Canis lupus*)	GrA-22501	14450	90	17923–17352	( Haesaerts, 2006, p. 38)
DW 2	AMS	Femur ( *Canis lupus*)	OxA-13044	14380	60	17815–17337	( Haesaerts, 2006, p. 38)
DW 2: Idem OxA-13044	AMS	Femur ( *Canis lupus*)	GrA-22094	14600	110	18181–17461	( Haesaerts, 2006, p. 38)
DW 4: OS 07-3 (1984) ( *Toptalishche*)	AMS	Femur ( *Canis lupus*)	SacA-14981	15210	130	18763–18252	( Haesaerts *et al.*, 2015, p. 385)
DW 1: OS 07-6 (1966)	AMS	Tibia ( *Vulpes lagopus*)	SacA-14982	14400	90	17886–17312	( Haesaerts *et al.*, 2015, p. 385)
DW 4: (To-2) KB-323, bord nord HA-4 (2005–2008)	Conventional	Femur ( *Mammuthus*)	GrN-29876	14550	70	18059–17435	( Haesaerts, 2006, p. 38)
DW 4: (To-2) KB-344, bord sud HA-4 (2005–2008)	Conventional	Femur ( *Mammuthus*)	GrN-29877	14560	70	18083–17450	( Haesaerts, 2006, p. 38)
DW 4: Idem GrN-29877	AMS	Femur ( *Mammuthus*)	OxA-15587	14790	60	18245–17928	( Haesaerts, 2006, p. 38)
DW 2, Pit 6: Z-2a upper (To-2) (2005–2008) MZH-5	AMS	Charcoal	SacA-11487	14600	60	18149–17540	( Haesaerts *et al.*, 2015, p. 385)
DW 2, Pit 6: Z-2a upper (To-2) (2005–2008) MZH-2	AMS	Charcoal	SacA-11486	14610	60	18162–17571	( Haesaerts *et al.*, 2015, p. 385)
DW 2, Pit 6: Z-2a middle (To-2) (2005–2008) OS 07-9	AMS	Bone ( *Mammuthus*)	GrA-38810	14750	50	18222–17907	( Haesaerts *et al.*, 2015, p. 385)
DW 2, Pit 6: Idem GrA-38810	AMS	Bone ( *Mammuthus*)	SacA-11177	14810	90	18266–17884	( Haesaerts *et al.*, 2015, p. 385)
DW 2, Pit 6: Z-2a middle (To-2) (2005–2008) OS 08-01	AMS	Metacarpal ( *Canis lupus*)	SacA-12040	15320	90	18820–18290	( Haesaerts *et al.*, 2015, p. 385)
DW 1, Pit 7: Z-2/3, OS 07-7	AMS	Femur ( *Mammuthus*)	GrA-38787	14590	60	18130–17515	( Haesaerts *et al.*, 2015, p. 386)
DW 1, Pit 7: Idem GrA-38787	AMS	Femur ( *Mammuthus*)	SacA-11176	15030	90	18647–18191	( Haesaerts *et al.*, 2015, p. 386)
DW 1, Pit 7: Idem SacA-11176	AMS	Femur ( *Mammuthus*)	SacA-14986	15430	90	18898–18337	( Haesaerts *et al.*, 2015, p. 386)
DW 1, Pit 8: Z-1 (To-1) OS 08-02	AMS	Rib ( *Mammuthus*)	SacA-12041	14830	90	18277–17891	( Haesaerts *et al.*, 2015, p. 390)
DW 1, Pit 8: Idem SacA-12041	AMS	Rib ( *Mammuthus*)	SacA-14984	14920	90	18620–17985	( Haesaerts *et al.*, 2015, p. 390)
DW 1, Pit 8: Idem SacA-12041	AMS	Rib ( *Mammuthus*)	SacA-12259	14970	90	18642–18106	( Haesaerts *et al.*, 2015, p. 390)
DW 1, upper cultural layer: TO-3A (2015)	AMS	Bone ( *Mammuthus*)	GrA-66076	14660	70	18212–17746	( Shydlovskyi *et al.*, 2017)
DW 1, upper cultural layer: TO-3B (2015)	AMS	Bone ( *Mammuthus*)	GrA-66078	14730	70	18221–17858	( Shydlovskyi *et al.*, 2017)

The initial age estimates (those with lab codes KI) were obtained using conventional radiocarbon methods and range from 24.1–14.6 ka cal BP. However, these broad ages are inconsistent with stratigraphic evidence suggesting the initial cultural layer predates the last Late Pleistocene loess accumulation cycle. Additionally, an outlier age of 16.1–14.6 ka cal BP (OXA-709), derived from a mammoth tooth excavated nearly 30 years before analysis, indicates a younger age. These ages contrast with more reliable ages from accelerator mass spectrometer (AMS) measurements that place the initial cultural layer within a narrower range of 15.4–14.3 ka uncal BP, corresponding to a calibrated age range of approximately 2000 years.

Analysing the calibrated AMS ages, Haesaerts identified three settlement phases corresponding to three cultural layers observed in the stratigraphic trenches of the 1 ^st^, 2 ^nd^, and 4 ^th ^Units ( Haesaerts *et al.*, 2015). Several layers within settlement features, such as pits and MBS 4, were associated with three distinct cultural phases identified across the entire settlement area:

1. The first phase (To1, Z1) spanned from 15,050–14,750 BP (18,300–17,900 cal BP; 400 years).
2. The second phase (To2, Z2) ranged from 14,900–14,500 BP (18,150–17,600 cal BP).
3. The third phase covers the period from 14,500–14,300 BP (17,750–17,400 cal BP).

These settlement phases indicate hiatuses of c. 200 years between each phase, with only the second and third phases associated with the presence of MBSs ( Haesaerts *et al.*, 2015). The identification of three cultural layers within MBSs 1, 2, and 4 suggests that each phase corresponded to a distinct Unit and MBS, implying at least three distinct episodes of habitation spanning c. 900 years ( Chabai *et al.*, 2020). Despite some rejected ages, these ages suggest synchronicity between Units 2 and 4 ( Haesaerts *et al.*, 2015).

However, such an interpretation relies primarily on mammoth bone ages that show significant temporal variation. Reliable mammoth bone ages range by millennia and may thus indicate a sustained use of scavenged mammoth remains following the LGM. Under such a scenario, the youngest mammoth bone ages mark the actual settlement period, possibly indicative of active hunting. This is corroborated by other non-mammoth samples, like charcoal and meso-mammal bones which indicate a significantly shorter period, forming two distinct groups: 18.2–17.3 ka cal BP and 18.8–18.3 ka cal BP.

Minimal age differences are observed between MBSs 1, 2, and 4, as well as between wolf and mammoth bones, with ages aligning with earlier mammoth material (18.2–17.3 ka cal BP), likely representing the main occupation period. Microstratigraphy comparisons between MBS 4 and Pit 6 show distinct differences but reveal concurrent use, as the ash layer age in Pit 6 matches the establishment of MBS 4. Multiple layers within MBS 4 suggest several habitation episodes.

In short, while identifying three settlement phases advanced understanding of the site, uncertainties remain about the duration of occupation. The correlation of lower pit layers with early settlement phases remains tenuous, and careful selection of samples is warranted as the stratigraphy of artificial objects may not align with the overall settlement sequence. The correlation of cultural layers in pits and MBS 4 with broader settlement layers raises concerns, as these features may not represent the overall stratigraphy. With that in mind, this study seeks an independent analysis of the stratigraphy of MBS 4 and Pit 6 and their immediate surroundings and radiocarbon data for more accurate comparisons.

## Materials and methods

In the absence of substantial charcoal samples, twelve meso-faunal osseous samples from three species ( *Vulpes sp., Lepus sp., Lupus sp*.) were selected from recent excavations with well-provenienced finds within cultural layers. Larger, well-preserved, compact bones (e.g. pelvis, long bones) were prioritised to minimise potential vertical displacement within the stratigraphy and maximise collagen extraction. One sample exhibited cutmarks, underscoring its anthropogenic accumulation.

The twelve samples were chosen from different archaeological contexts within the site. Four samples originated from archaeological layers within MBS 4, highlighting different aspects of its occupation. Another four samples were derived from Pit 6, to clarify its cultural accumulation and activity. The remaining four samples were selected from saturated cultural layer surrounding MBS 1 (i.e., *Toptalishche)*, providing a broader understanding of the wider site use.

AMS dating was employed on twelve samples after pre-treatment with an acid-base-acid (ABA) protocol following the standard protocols of the Centre for Isotope Research at Groningen University ( Dee *et al.*, 2020). High-precision measurements of δ ^13^C and δ ^15^N were also conducted prior to AMS measurement to assess sample suitability.

Chronological modelling was undertaken using OxCal v4.4 using the IntCal20 calibration dataset to refine estimates for the site’s chronology ( Ramsey, 2017; Reimer *et al.*, 2020). A Kernel Density Estimation (KDE) in combination with Bayesian start/end date modelling was used to summarise the distributions of each occurrence based on the available chronological data incorporating 5 previously obtained AMS ages from Pit 6 for a total of 16 ages ( Haesaerts *et al.*, 2015). Radiocarbon age determinations inherently contain a degree of uncertainty expressed as a distribution of radiocarbon ages. KDE accounts for this uncertainty by sampling individual radiocarbon age ranges to generate a set of probable calibrated age ranges for each event within a given dataset. The algorithm then applies a KDE to these sampled ages, producing a smoothed estimate of the temporal event density over 10,000 iterations and averaging the results.

This approach enables the segregation of phases, represented here by the different localities from which the samples were obtained (i.e., MBS 4, *Toptalishche,* Pit 6) and subphases, denoted by the cultural layers within these localities (i.e., Z2 and Z3 within Pit 6 and To3/2 and T3 within the *Toptalishche*).

## Results

11 new AMS radiocarbon age determinations were obtained ( Table 3), along with the associated analytical data for each of the dated bone samples. Of the 12 samples analysed, 11 produced usable collagen, with elemental values (%C, %N) falling within the accepted ranges for well-preserved collagen ( Talamo *et al.*, 2021). The one failed sample, from Unit 1, To-3, V20#108, contained insufficient collagen to meet the minimum reliable threshold and was therefore excluded from further analysis. Among the remaining 11, the atomic C/N ratios for all samples ranged from 3.1–3.3, within the acceptable limits of 2.9–3.5 ( Higham *et al.*, 2011). %C yields were high, ranging from 43.6%–39.8%.

**Table 3.** Radiocarbon ages and isotopes of newly obtained samples.

Unit, context	Dated material *taxa; element); * *notes*	Lab-ID	F ^14^C	± 1- sigma	^14^C Age (yrBP)	± 1- sigma	Calibrated age (95.4% probability)	Yld (%)	%C	%N	C/ N	δ ^13^C (‰;IRMS)	± 1- sigma	δ ^15^N (‰;IRMS)	± 1- sigma	Surface elevation (m)
Unit 1, To-3/2, V19, #532	Collagen (small/medium sized mammal; radius?)	GrM-31355	0.1687	0.0008	14295	40	17510– 17108	1.1	43.0	15.7	3.2	-21.31	0.15	7.28	0.30	-4.38
Unit 1, To-3, V20, #31	Collagen (small/medium sized mammal; phalange)	GrM-31356	0.1595	0.0007	14745	40	18216– 17918	0.5	42.7	15.5	3.2	-18.92	0.15	7.82	0.30	-4.28
Unit 1, To-3, V20, #53	Collagen ( *Vulpes*; pelvis)	GrM-31358	0.1609	0.0008	14675	40	18174– 17840	0.9	42.4	15.2	3.3	-20.65	0.15	1.86	0.30	-4.29
Unit 1, To-3, V20, #108	Collagen (failed) ( *Lepus*; scapula)	N/A	N/A	N/A	N/A	N/A	N/A	0.2	N/A	N/A	N/A	N/A	N/A	N/A	N/A	-4.31
Unit 2, Pit 6, Z-2, 23A, #838A	Collagen ( *Vulpes*; femur); Glue paraloid	GrM-31359	0.1628	0.0008	14580	40	18059– 17533	0.9	41.0	14.8	3.2	-19.35	0.15	3.80	0.30	-4.84/-4.89
Unit 2, Pit 6, Z-2, 23A, #838B	Collagen ( *Vulpes*; vertebrae)	GrM-31374	0.1598	0.0007	14730	40	18209– 17900	6.3	42.6	15.4	3.2	-19.27	0.15	4.26	0.30	-4.84/-4.89
Unit 2, Pit 6, Z-2, 23B, #444	Collagen (small/medium sized mammal; molar)	GrM-31360	0.1593	0.0007	14760	40	18225– 17933	4.6	43.6	15.7	3.2	-19.46	0.15	9.33	0.30	-4.88
Unit 2, Pit 6, Z-3, 23A, #1106	Collagen ( *Lupus*; phalange I)	GrM-31361	0.1629	0.0008	14580	40	18059– 17533	9.5	42.0	15.0	3.3	-18.84	0.15	8.48	0.30	-4.33
Unit 4, Dw 4, 332Б, #57	Collagen ( *Lepus*; pelvis)	GrM-31364	0.1624	0.0008	14600	40	18131– 17596	4.4	40.9	14.6	3.3	-20.5	0.15	0.97	0.30	-4.01
Unit 4, Dw 4, 333A, #1	Collagen ( *Lupus*; phalange I)	GrM-31365	0.1590	0.0008	14775	40	18231– 17954	9.3	41.1	14.8	3.2	-19.37	0.15	6.10	0.30	-4.03
Unit 4, Dw 4, 333A, #2	Collagen ( *Lupus*; metatarsus III)	GrM-31366	0.1603	0.0007	14705	40	18193– 17872	7.5	39.8	14.4	3.2	-19.38	0.15	6.31	0.30	-4.05/-4.06
Unit 4, Dw 4, 334A, #161	Collagen ( *Lepus*; femur) ; Glue paraloid	GrM-31367	0.1587	0.0008	14790	40	18239– 17970	2.0	41.6	15.0	3.2	-20.55	0.15	1.66	0.30	-4.11/-4.12

The results of the KDE Bayesian Start-End model (SI1) show that the ages from small mammals from the selected site features can be constrained from 18,504–18,238 years cal BP to 17,865–17,431 years cal BP with a site duration lasting between 396–1027 years.

This model had an agreement of A=51.3%, below the standard accepted agreement index of A'c= 60.0%. This poor agreement can be attributed to two outliers. The first, is GrM-31355 (Unit 1, To-3/2, V19, #532) where the agreement index is low (A=6.7%). This is anomalous because it is from To-3/2 and should therefore be found at least 9 cm deeper in the sequence of the *Toptalishche* than the other ages from the *Toptalishche*. This bone is a possible radius of a small/medium sized mammal though it appears to be gnawed or weathered, and its position may therefore be the result of either anthropogenic (e.g., trampling) or nonanthropogenic (e.g., burrowing) mixing of the *Toptalishche*.

Another age not in agreement is that of SacA-12040 (DW 2, Pit 6: Z-2a middle (To-2) (2005–2008) OS 08-01) where A=58.2%, just below the agreement is 60%. The sample is a *Canis lupus* metacarpal. It is difficult to explain why this age is older than the age of a charcoal sample from the same layer (DW 2, Pit 6: Z-2a middle (To-2) (2005–2008) OS 08-01) which is good agreement with the model.

**Figure 5.**
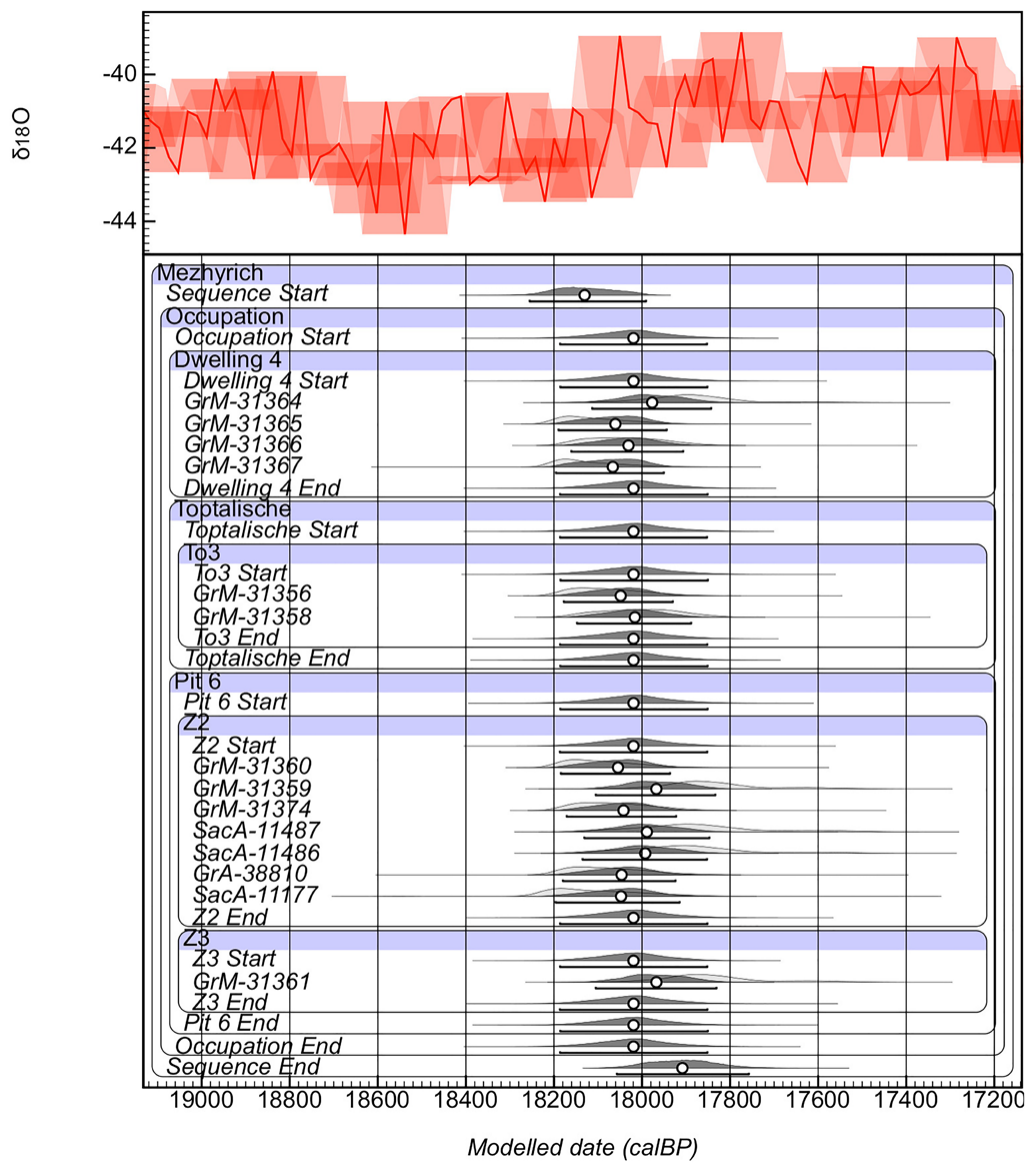
Results of the KDE Bayesian Start-End model of the radiocarbon ages.

As a result, the model was rerun excluding the two outliers resulting in high agreement indices ( Figure 5; A _model_=91.9%, A _overall_=70.5%). The model shows that the ages from small mammals from the selected site features can be constrained from 18,248–17,992 years cal BP to 18,056–17,764 years cal BP with a site duration lasting between 0–429 years (SI2). The ages of the individual features can be further constrained with MBS 4 occurring between 18,184–17,856 to 18,185–17,862 years cal BP (span=0–320). The *Toptalishche* (To3) is constrained between 18,182–17,856 years cal BP (span=0–368). Pit 6 starts between 18,185–17,857 to 18,181–17,855 years cal BP (span=0–392). The layer within Pit can be further subdivided: Z2 is constrained to between 18,184–17,857 to 18,185–17,858 years cal BP (span=0–343) while Z3 is constrained to 18,185–17,857 to 18184–17859 years cal BP (span=0–279).

## Discussion

This study has augmented the chronological framework for the Mezhyrich MBS 4 site, including features such as Pit 6 and the *Toptalishche*, through new radiocarbon dating of faunal remains from cultural layers. The new results provide a refined understanding of the temporal dynamics of the site’s occupation, constraining the chronology to less than 429 years, excluding two outliers. The high overall fit of our model, which incorporates previous AMS ages, justifies excluding these outliers from our analysis.

### Occupation frequency and duration of MBS 4

A primary objective was to determine the frequency and duration of occupations at Mezhyrich DW4. Compared to previous ages from MBS 4 and its surrounding area, the conventional ages obtained from the Kyiv laboratory were clear outliers in the chronostratigraphy. Later AMS ages, however, narrow the habitation timeframe to 18,296–17,486 years cal BP ( Shydlovskyi *et al.*, 2023), and the newly obtained ages for the middle cultural layer align with this revised timeframe with modelling further refining them.

The ages from cultural contexts show significant overlap, rendering it impossible to identify potential hiatuses in occupation. Specifically, the MBS 4 layers are attributed to 18,184–17,862 years cal BP, with no chronological evidence of gaps in occupation and a duration of less than 320 years. Despite this, stratigraphic evidence points to three distinct horizons in the MBS and pit areas. These occupations may have been brief, possibly lasting weeks, or involved multiple reoccupations over years, as suggested by the thinness of associated deposits. No clear temporal trend emerged among the MBS units, implying they were repeatedly and contemporaneously used, with potential relocations over time.

Our revised chronology does not unequivocally differentiate discrete occupational phases; rather, it suggests that these occupations were penecontemporaneous from a radiocarbon perspective. However, emerging evidence strongly indicates that skeletal remains of long-deceased individuals were deliberately scavenged and incorporated into site construction. This phenomenon, also documented at Kostenki 11-Ia approximately 7,000 years earlier, implies a persistent cultural tradition of reusing human remains in architectural contexts ( Rey-Iglesia *et al.*, 2025).

### Chronology of Pit 6 (Unit 2)

The chronology of Pit 6 reveals complexities, with layers Z2 and Z3 showing indistinguishable ages despite a 56 cm difference in depth, likely due to layer sloping. The outlier from Z2 (DW 2, Pit 6: Z-2a middle) further complicates the picture. Radiocarbon ages for Z-2 range from 15.3–14.6 ka uncal BP, corresponding to 18.8–17.5 ka cal BP, with the most reliable ages derived from charcoal samples. These narrow the timeframe of the second phase of the site’s occupation to 18.0–17.6 ka cal BP ( Haesaerts *et al.*, 2015; Shydlovskyi *et al.*, 2020; Shydlovskyi *et al.*, 2023).

Notably, two distinct age clusters emerged within Z-2: one spanning 14.810–14.730 ka uncal BP and the other 14.610–14.580 ka uncal BP, separated by a 120-year gap. However, artefacts from both clusters were found at the same depth, casting doubt on the correlation between radiocarbon ages and stratigraphic layers. Given the improbability of a pit maintaining integrity over such a long period without substantial alteration, it seems unlikely that the age range reflects the true chronology of Pit 6.

### Chronology of the Toptalishche

In the Toptalishche, the youngest age (MZH, Unit 1, To-3/2) from the transitional zone between cultural layers 2 and 3 may indicate a post-occupation event and is excluded from further analysis. Previous studies obtained two ages from mammoth bone fragments in the upper cultural layer (To-3) south of MBS 1, which range from 14,730–14,660 uncal BP (18,221–17,746 cal BP; Shydlovskyi *et al.*, 2023). However, a bone from beneath the primary cultural layer yielded a younger age of 14,295 uncal BP (17,510–17,108 cal BP), raising concerns about its stratigraphic position, possibly due to post-depositional processes like rodent activity.

Unit 1 presents two distinct dating clusters, with the first ranging from 14,745–14,660 uncal BP and the second from 14,450–14,295 uncal BP ( Shydlovskyi *et al.*, 2023). Mammoth bones from Pit 7 and Pit 8, associated with the same Unit, show older ages, possibly reflecting ancient collection practices. This consistency across various features suggests the active procurement of mammoth bones by settlement inhabitants, though dating mammoth bones may not accurately reflect operational periods ( Shydlovskyi *et al.*, 2022).

### Correlating internal layers of MBS 4 to external features

To resolve the synchronicity/asynchrony between MBS 4 and other site features, we propose viewing the objects within each Unit as functioning synchronously within specific periods, forming components of a single settlement module. Although individual features may have unique histories, the spatial clustering around MBS Units suggests a cohesive economic group. Thus, the pit layers cannot be isolated from the MBS Units in the same context. Radiocarbon ages of MBS 4, the *Toptalishche*, and Pit 6 cluster around the same timeframe, though Pit 6 shows a wider distribution, possibly due to the greater number of ages sampled. This clustering explains the significant variance in mammoth bone dating, with settlement ages from mammoth bones ranging widely between 15,400–14,300 uncal BP (18,900–16,600 cal BP), without forming visible peak values ( Shydlovskyi *et al.*, 2022).

### Isotopic analysis

**Figure 6.**
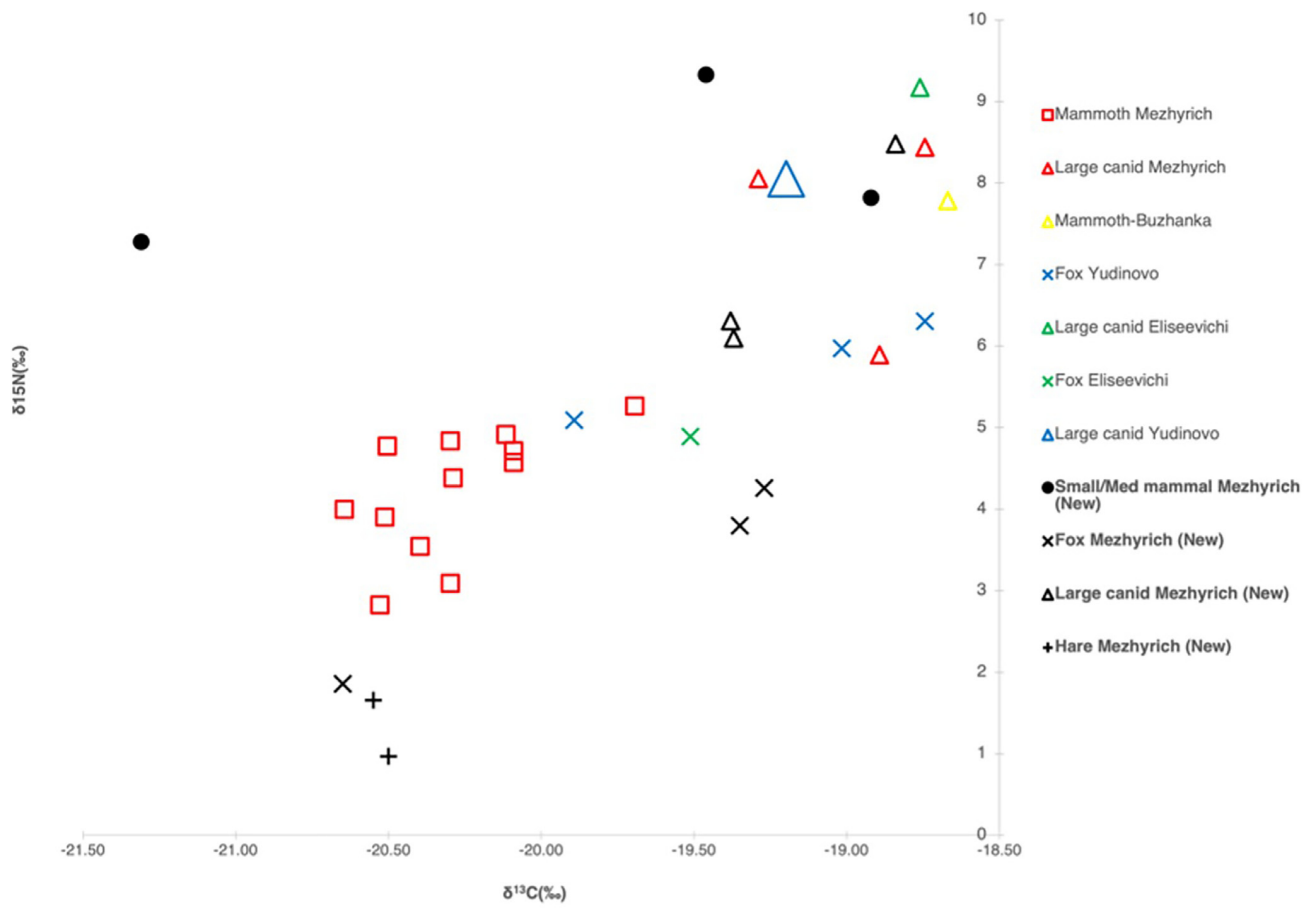
Stable isotopes of identified taxa from Mezhyrich compared to those from Drucker *et al.* (2018). Bolded text indicates newly presented samples.

Isotopic data from identified taxa, as shown in Figure 6, show that isotope values plot similarly to those of previous studies of similar time frames and contexts ( Drucker *et al.*, 2018). Still, large canids ( *C. Lupus*) from MBS 4 have slightly lower δ ^15^N values than previous measurements from Mezhyrich. Two fox samples cluster around earlier measurements, while one sample (Unit 1, To-3, V20, #53) plots low, comparable to the δ ^15^N values of hare species. A single hare sample also exhibits very low δ ^15^N, suggesting the need for further investigation into δ ^15^N depletion or a reassessment of taxonomic attribution.

### Site function

It has been observed that basic features as MBSs, production centres, hearths and storage pits are always located in a certain relationship, thus forming distinct functional *Units* ( Shovkoplias, 1971), i.e., spatial (settlement) modules consisting of the remains of the MBS itself together with other household features around it. As a result of many years of research, the remains of four Units were discovered on the Mezhyrich settlement – four MBSs around which functionally differentiated features are located.

Others have suggested that MBSs may not have always functioned as dwellings. MBSs at sites like Anosovo, Mizyn and other similar structures, may not reflect dwellings. Rather, in these cases, the mammoth bones were likely deliberately laid over the site before being covered with soil, marking the end of a settlement's occupation, a pattern possibly influenced by religious ideas ( Gavrilov, 2024; Sablin *et al.*, 2023). Other structures, such as those at Kostenki 11 have been suggested to have been too large to have been covered dwelling structures and the relatively sparse occurrence of domestic tools may suggest other functions ( Pryor *et al.*, 2020). Even among MBS site complexes where MBSs are thought to have served potentially different functions, the main criteria for distinguishing dwelling structures from other functional units are only assumed by the inclusion of associated features (e.g., artificial hollows, hearths, and cultural layers inside and outside of the structures ( Zheltova, 2024). In such cases, others have suggested that a main way of identifying dwelling structures is through multiproxy analysis of the faunal remains ( Djindjian, 2015).

While MBSs may have served different functions at other sites, and even possibly within Mezhyrich, our new chronological model lends support to the body of evidence suggesting that MBS 4 functioned primarily as a dwelling structure. The large amount of domestic activity testified by lithics and worked animal remains found both within and in associated features of MBS4, all tightly constrained around a timeframe of less than 429 years with distinct activity layers suggests that MBS 4 was a site that was used and returned to at a generational scale.

Thus, here, the Unit is a spatial settlement module consisting of the remains of the MBS itself together with the household objects around it and is the result of dwelling and other domestic activities.

## Conclusion

New radiocarbon ages from Mezhyrich MBS4 and its surrounding features enhance our understanding of Late Pleistocene human activity at Mezhyrich, emphasising the importance of sampling strategy to interpret archaeological data. The newly derived chronology is consistent with a single occupation model, but cannot exclude repeated occupation within a maximum of several centuries around 18 ka cal BP. This provides insights into settlement dynamics and the use of MBSs in Eastern Europe and contributes to broader discussions about Late Pleistocene life in the East-Central European Plain.

Given the constant refinement of the radiocarbon dating, and the fact that the most recent ages originate from material with well-documented provenance from recent excavations, most of these ages likely reflect a duration of the settlement’s existence. However, while KDE modelling effectively summarises the available chronological information, it does not necessarily provide an accurate reflection of through-time variation in occurrence counts. Peaks and troughs in the density distribution may not reliably represent actual increases or decreases in the occurrence of events over time, as radiocarbon datasets inevitably constitute an incomplete sample of the underlying phenomenon. As a result, the precise positions of the oldest (lowest) layers across various features remain uncertain.

Thus, future radiocarbon ages should include:

- Collecting a series of samples from the fill of Pit 6, layer Z-1.
- Obtaining samples from both the lower and upper layers of MBS 4’s fill.
- Acquiring more pertinent samples from the lower layer of the *Toptalishche*.

In scenarios involving differences in the settlement’s visitation stages by the prehistoric collective (i.e., anthropogenic layers) spanning several years or within a single generation, we underscore that correlating these with the site’s radiocarbon chronology will be exceedingly challenging, given the inherent methodological limitations in accuracy. For a further refined assessment with Bayesian modelling, it would be valuable to have detailed information on the stratigraphic provenance of the aged samples alongside a robust understanding of the site’s post-depositional processes.

Further research and analysis, incorporating a variety of dating techniques and multi-proxy sedimentary analysis focusing on depositional taphonomy, are needed to gain a more accurate understanding of the site’s occupation and the duration of specific episodes within individual Units. Here, additional radiocarbon dating, focusing on charcoal samples and bones from smaller mammals, is crucial. By considering mammoth bone dating characteristics and integrating chronological information with the stratigraphy and spatial grouping of objects, a more nuanced interpretation of the site’s history and its significance in the context of prehistoric human activity can be achieved.

## Ethics and consent statement

Ethical approval and consent were not required.

## Data Availability

All radiocarbon ages analysed during this study are included in the main article along with their associated data. No additional datasets were generated or analysed during the current study.
