# Supplementary material for: A revised radiocarbon chronology for the mammoth bone structures and associated features at Mezhyrich, Ukraine
Source: Open Res Eur. 2025 Jul 25;5:198. [Version 1] doi: 10.12688/openreseurope.20112.1 (PMC12639289; doi:10.12688/openreseurope.20112.1)
Supplement: Peer review history

# Reviewer report: Reviewer response for version 1

**DOI:** 10.21956/openreseurope.21756.r57264

**Published:** 2026-01-19

**Authors:** Natasha Reynolds

**Affiliations:** 1 Universite de Bordeaux, Talence, Nouvelle-Aquitaine, France

Chu et al. (2025) present new radiocarbon results for the important Late Upper Palaeolithic site of Mezhyrich, Ukraine, and discuss their implications for the interpretation of the site, especially concerning phases of activity.

 The site of Mezhyrich is best known for its remarkable mammoth bone structures. These structures, found at multiple sites in Eastern Europe, have usually been seen as the remains of dwelling structures. In recent years this interpretation has been subject to increased debate – not simply, as Chu et al. state, because “most were excavated decades ago”, but because new critical attention has been paid to the arguments that underlie this interpretation, including the taphonomy of the structures and the feasibility of constructing dwellings, sometimes many metres in diameter, from mammoth remains (e.g. Gavrilov 2015). New data concerning chronology, seasonality and faunal assemblage compositions has also helped to improve our understanding of these structures (e.g. Pryor et al. 2020, Sablin et al. 2025, Rey-Iglesia et al. 2025).

 Our overall knowledge of the chronology of Late Upper Palaeolithic Eastern Europe sites remains poor, and is often reliant on radiocarbon dates of dubious accuracy. Thanks to previous work at Mezhyrich (Haesaerts et al. 2015), this site was actually already relatively well-dated, but the obtention of new dates is still very welcome.

 However, there are some problems with the current version of the paper that I believe need attention from the authors. First, there is a problem of over-interpretation of radiocarbon dating results and ignoring of error estimates. This is seen particularly in the section on Chronology of Pit 6 (Unit 2). It is not appropriate, statistically, to search for “clusters” within a very small number of dates which apparently all overlap at 95.4% probability once calibrated. The authors state that “Notably, two distinct age clusters emerged within Z-2: one spanning 14.810–14.730 ka uncal BP and the other 14.610–14.580 ka uncal BP, separated by a 120-year gap.“ This is only the case if you entirely ignore the error estimates that are attached to these ages. No statistical testing of these “clusters” was carried out.

 Through the paper there is a problem of treating radiocarbon age estimates as precise point values, rather than as probability distributions. The real age of the samples will only rarely match the median of the distribution. Furthermore, radiocarbon ages should always be cited with their error estimates – these are omitted throughout the paper. More care also needs to be taken in the citing of “ranges” of radiocarbon dates – again, it’s not appropriate to ignore the error values when giving a range of dates.

 The second problem requires a different type of attention. In the final sections of the paper, the authors write that “While MBSs may have served different functions at other sites, and even possibly within Mezhyrich, our new chronological model lends support to the body of evidence suggesting that MBS 4 functioned primarily as a dwelling structure.” However, there is no argument developed anywhere in the paper to support this conclusion. As mentioned above, the current debate on the interpretation of mammoth bone structures has numerous axes, including taphonomy and feasibility. However, this paper does not engage with any of these aspects of the debate. The authors suggest that their dating work “lends support” to the interpretation of MBS 4 as a dwelling structure, but it is not clear how it lends support to this interpretation over any other possibilities: for example, as suggested for other sites, that it was a ritualised midden (Sablin et al. 2025) or could have been used for food storage (Pryor et al. 2020). In any case it is not clear to me how chronological work alone could be used to distinguish between such possibilities. In order to argue for one interpretation over another, further arguments beyond chronology need to be presented. These are absent from this paper, meaning that there is a disconnect between the results of the chronological study and the paper’s conclusions.

 There are a few other points of varying degrees of significance that the authors might like to consider:

 1. What is the evidence for the mammoth remains having “likely been primarily obtained through scavenging”? Chu et al. write that “The mammoth bones used in the construction of MBS 4 were sourced from a minimum of 37 individuals, predominantly from natural accumulations, though some were obtained in anatomical position as evidenced by at least one freshly deceased mammoth carcass (Gladkih et al., 1984).” However, in the cited paper by Gladkih et al., it is only stated that "The bones from the different carcasses vary greatly in their degree of preservation. Indeed, the variation is so great that it suggests *some *[my emphasis] of the bones were picked up from skeletons of long-dead animals." This is quite different from saying that the bones were “predominantly from natural accumulations”.

 2. Numerous recent papers on the debate around mammoth bone structures are not cited here – for example, Gavrilov (2015), Sergin (2019), Khlopachev & Gavrilov (2019), Khlopachev (2021), Oliva (2021).

 2. Background section – omits the contribution of Rogachev (1962) to the development of the dwellings interpretation.

 3. “meso-faunal osseous samples from three species (Vulpes sp., Lepus sp., Lupus sp.)” – the text should make it clear that some samples were from non-identified species. It’s a pity that ZooMS wasn’t used to make determinations in these cases.

 4. “small mammals” – although it’s a little outside my area of expertise, I think in zooarchaeology this term typically refers to animals that are considerably smaller than wolves or foxes (e.g. rodents). This usage may therefore be confusing to the reader.

 5. Figure 5 – It should be made clear in the caption that this is the version of the model that was run excluding outliers.

 6. “reusing human remains in architectural contexts” – this wording is unfortunately confusing, as it suggests that human remains (rather than mammoth remains) were included in the structure. The use of “individuals” in the preceding sentence of the manuscript is also unclear – it’s not obvious at a glance that it refers to mammoths.

 Although this review could not be as positive as I would have liked it to be, I remain very glad to see new research on these structures. I hope that the authors will be able to take these comments in the constructive manner they are intended, and develop their arguments further. I very much look forward to further contributions from them as their research continues.

Is the study design appropriate and does the work have academic merit?

Partly

Is the work clearly and accurately presented and does it cite the current literature?

Partly

If applicable, is the statistical analysis and its interpretation appropriate?

Partly

Are all the source data underlying the results available to ensure full reproducibility?

Yes

Are the conclusions drawn adequately supported by the results?

Partly

Are sufficient details of methods and analysis provided to allow replication by others?

Yes

Reviewer Expertise:

Upper Palaeolithic archaeology of Europe, especially Russia; radiocarbon chronology; lithic analysis; archaeological theory; Soviet and post-Soviet archaeological history; mammoth bone structures

I confirm that I have read this submission and believe that I have an appropriate level of expertise to state that I do not consider it to be of an acceptable scientific standard, for reasons outlined above.

## References

[1] : A revised radiocarbon chronology for the mammoth bone structures and associated features at Mezhyrich, Ukraine. *Open Research Europe* .2025;5: 10.12688/openreseurope.20112.1 10.12688/openreseurope.20112.1 41280290 PMC12639289

[2] : Contribution à la stratigraphie du site paléolithique de Mezhyrich (Ukraine). *L'Anthropologie* .2015;119(4) : 10.1016/j.anthro.2015.07.002 364-393 10.1016/j.anthro.2015.07.002

[3] : Les cabanes de type « Anosovka-Mézine » du site de Yudinovo : éléments de construction, architecture, classification. *L'Anthropologie* .2021;125(4) : 10.1016/j.anthro.2021.102923 10.1016/j.anthro.2021.102923

[4] : Mammoth bone structures: A Comparison of Dolní Věstonice and Spadzista Street Site in Kraków. *L'Anthropologie* .2021;125(4) : 10.1016/j.anthro.2021.102917 10.1016/j.anthro.2021.102917

[5] : The chronology and function of a new circular mammoth-bone structure at Kostenki 11. *Antiquity* .2020;94(374) : 10.15184/aqy.2020.7 323-341 10.15184/aqy.2020.7

[6] : Ancient biomolecular analysis of 39 mammoth individuals from Kostenki 11-Ia elucidates Upper Palaeolithic human resource use. *Quaternary Environments and Humans* .2025;3(1) : 10.1016/j.qeh.2024.100049 10.1016/j.qeh.2024.100049

[7] : The Epigravettian Site of Yudinovo, Russia: Mammoth Bone Structures as Ritualised Middens. *Environmental Archaeology* .2025;30(1) : 10.1080/14614103.2023.2182977 50-70 10.1080/14614103.2023.2182977

[8] : “Dwellings” of the Anosovo-Mezin Type: Origins and Interpretation. *Stratum plus.* .2015. No.1;

[9] : Mammoth-Bone Dwellings on the Russian Plain. *Scientific American* .Vol. 251, No. 5 (November 1984) pp. 164-175; Reference source

[10] : Paleoliticheskie zhilishcha anosovsko-mezinskogo tipa: Konstruktivnye osobennosti i problema interpretatsii. *Rossiiskaia Arkheologiia* .2019 (4): 27–42.; Reference source

[11] : Dwellings or sanctuaries?. *Stratum plus* .2019, 99-110; Reference source

[12] : Ob anosovsko-mezinskom tipe paleoliticheskikh zhilishch na Russkoi ravnine. *Kratkie Soobshcheniya Instituta Arkheologii* .1962; 92: 12–17; Reference source

# Reviewer report: Reviewer response for version 1

**DOI:** 10.21956/openreseurope.21756.r62139

**Published:** 2025-11-21

**Authors:** Andrzej Wiśniewski

**Affiliations:** 1 University of Wrocław, Wrocław, Poland

The article addresses several very important issues: 1. assessment of the age of MBS4 and assessment of the chronological relationship between cultural layers from MBS and peripheral structures.The authors answer the questions posed in accordance with current methods, providing very important results that clarify the chronology of the analysed objects and the time of their use. I have no substantive comments. I rate the article as very excellent.

 I have only a few grammatical comments regarding the spelling of names in references, and in one case the full title of the publication needs to be provided. See the list below.

 1. Dee MW, Palstra SWL, Aerts-Bijma AT, et al.: Radiocarbon dating at **groningen**

 2. Gaudzinski-Windheuser S: An introduction to living structures and history

 of occupation at the Late **upper paleolithic** site of Oelknitz

 3. Pidoplichko IG, Allsworth-Jones P: Upper **palaeolithic**

 4.Rekovets L, Nowakowski D, Lech K: Analyse des micromammifères du site

 épigravettien de Mezhyrich (Ukraine). L’Anthropologie. 2014 - Complete the title of the publication.

 I would suggest correcting Figure 3. The hearts should be more prominent. It looks as if the figure was drawn by hand, especially the pits and Toptalishche structures are strangely marked.

Is the study design appropriate and does the work have academic merit?

Yes

Is the work clearly and accurately presented and does it cite the current literature?

Yes

If applicable, is the statistical analysis and its interpretation appropriate?

Yes

Are all the source data underlying the results available to ensure full reproducibility?

Yes

Are the conclusions drawn adequately supported by the results?

Yes

Are sufficient details of methods and analysis provided to allow replication by others?

Yes

Reviewer Expertise:

Palaeolithic archaeology, Geoarchaeology, Technology, Settlement strategies

I confirm that I have read this submission and believe that I have an appropriate level of expertise to confirm that it is of an acceptable scientific standard.

# Reviewer report: Reviewer response for version 1

**DOI:** 10.21956/openreseurope.21756.r62138

**Published:** 2025-11-21

**Authors:** William Chase Murphree

**Affiliations:** 1 Universidade do Algarve, Faro, Faro District, Portugal

This paper is focused on providing a revised chronology for the late Pleistocene Mammoth bone structure (MBS) 4 from Mezhyrich (Cherkasy Oblast, Ukraine). The goals of this paper were to better understand the occupational history of MBS 4. Using new ^14^C results, the authors have provided a new short chronological framework for the occupations of MBS 4 and it's surrounding features to a short window of around 429 years. 

 Generally, I thought the paper is well written, very interesting, and has a solid structure. However, I do have some specific comments and questions regarding the stratigraphic context of these samples. I also have some minor comments as well. I hope my comments and suggestions are constructive for the authors and fixable.

**Main comments:**

 Overall, I found the descriptions of the context of the samples and stratigraphic descriptions are lacking and hard to follow throughout the text, especially in regards to MBS 4. Considering how important context is to the premise of this paper and its conclusions, this issue is problematic but fixable. 

 Starting in the background chapter, I would suggest a full revision of Figure 4. It is unclear what the section photograph on the right is referring to, the current version is missing key points of overlap with the section drawing. I would suggest adding labels on the photograph indicating the layers to improve the readability of the figure as well as adding markers to show the square ID, especially if the photo is  from the same location as the drawing. If the two images are not from the same location then they should be labelled as such. Otherwise both described in detail in the caption. 

 Something that I feel that is relatively ambiguous throughout the discussion and tables is the stratigraphic context for the samples from MBS 4 (Unit/ DW 4). This information is not included in Table 3 and is glossed over in the discussion. The authors only provide a relative height from surface in Table 3. The other samples from Pit 6 and the toptalishche have layer designations. This comes off as confusing to me as the authors repeatedly refer to three distinct layers within MBS 4 (Unit/DW 4) and the importance of understanding the occupation history throughout the text but then do not state from where the samples are coming from. I think it would help improve the discussion or conclusion if they would provide more detail on layers or relative position the samples are taken from in Table 3 and in Figure 4 or if this data is not available state this more explicitly in the text. It's understandable that samples maybe have been procured from an earlier excavation or isn't available but I believe that should be explicit in the text. Additionally, in the discussion section for MBS 4, I don't quite understand how occupations can be both contemporaneous and yet be in stratigraphically distinct horizons. I think the phrasing of this section should be clarified. 

**Reproducibility:**

 Currently, there is no code available with this paper. The authors also mention SI2 (pg. 11) in the results, but it is currently unavailable online.  

**Minor comments:**

 1. Within the text and tables the authors occasionally alternate between using MBS and DW without giving a clear definition for each phrase. It a bit difficult to follow through the text. I would suggest these terms be defined simply in the background section to improve the readability throughout the rest of the text.  

 2. Section on how units are defined should be in the background as well. I understand the authors choice to include this section in the discussion but prior to this section it was unclear if unit referred to the MBS/DW or excavation area in which they were found. Possibly they could include a sentence or two in the background section to clarify. 

 3.  In the Chronology section, the three cultural layers/ phases is a bit confusing with how it's worded. All three phases have some overlap in terms of dating so the hiatuses between phases isn't very clear in the current text.

 4. Following up on the three phases, It also is not clear what the authors meant by three cultural layers within the MBSs. Does this suggest that within the last two phases there are multiple sublayers within the individual MBSs? I think this section should be revised for greater clarity.

Is the study design appropriate and does the work have academic merit?

Yes

Is the work clearly and accurately presented and does it cite the current literature?

Yes

If applicable, is the statistical analysis and its interpretation appropriate?

I cannot comment. A qualified statistician is required.

Are all the source data underlying the results available to ensure full reproducibility?

Partly

Are the conclusions drawn adequately supported by the results?

No

Are sufficient details of methods and analysis provided to allow replication by others?

Partly

Reviewer Expertise:

Geoarchaeology; Microstratigraphic analysis

I confirm that I have read this submission and believe that I have an appropriate level of expertise to confirm that it is of an acceptable scientific standard, however I have significant reservations, as outlined above.

# Reviewer report: Reviewer response for version 1

**DOI:** 10.21956/openreseurope.21756.r61253

**Published:** 2025-10-28

**Authors:** Timothy J Heaton

**Affiliations:** 1 University of Leeds, Leeds, England, UK

**A revised radiocarbon chronology for the mammoth bone structures and associated features at Mezhyrich, Ukraine**

**19 ^th^ October 2025**

**Summary**

 The paper provides new ^14^C dates and a re-analysis of an interesting archaeological site in Mezhyrich (Cherkasy Oblast, Ukraine) where mammoth bone structures from the Late Pleistocene have been discovered. Specifically, the authors have:

1. Provided a set of 11 new AMS ^14^C dates on samples from the site (Units 1, 2 and 4)
2. Combined these with existing AMS dates from the same units obtained by others
3. Fitted an updated chronological model using OxCal and a KDE modelling approach   

I felt the paper is very nicely written. It provided a very engaging and readable story. I enjoyed reviewing it, and it certainly taught me new things.   

 I do however have some specific questions about the modelling approach (and the need for a KDE/non-parametric approach). I hope/think that these queries can be addressed by the authors. I have written a fair bit below (but this is mainly for explanation). I hope my actual suggestions for edits to address these queries (given in the appropriate section) are relatively simple/quick.

 I hope my comments/suggestions are useful and constructive. 

**Main Review Comments on Modelling**

 My specific queries related to the modelling are provided below:

**Do we need a KDE approach?**

 The authors say that they use a KDE modelling approach in their analysis. However, it is unclear what this corresponds to and critically what it means. The model presented in Fig 5 appears to have some internal phases/groupings (MBS 4, Toptalische, Pit 6) some of which have further phases/subgroupings. Within each grouping there are then the individual ^14^C samples. What dates exactly are being modelled non-parametrically (all the dates; the dates within each group; the start/end of each group/sub-group)? And why is this needed?

 To me, incorporating a KDE model into a begin/end phase approach makes it very unclear both what the prior information actually incorporated into the model is, and more importantly what effect it will have on the resultant inference.

 In my personal opinion, the KDE model here seems rather unnecessary – especially when you have a lot of genuine prior information and not that many dates. Is there a reason why a much more traditional uniform phase type model isn’t more useful and appropriate – not least in terms of clarity of interpretation and explanation?

**Outliers in a KDE modelling approach**

 Related to the above, I do not understand what an OxCal agreement score would mean (or how it should be interpreted) in a context where you have specifically aimed to place no assumption on the form of the calendar age distribution between the start/end dates (by using a KDE). How can you tell if something is an outlier in such a context?

 This score really just appears to flag those two samples which determine the start/end of the (non-parametric) phase. By removing these you are substantially shrinking the range of the model (i.e., and hence your interpretation). However, under a KDE model I am not sure you really have solid grounds to use agreement to identify such outliers.

 I do not believe you can get reliable/quantitative information on which samples are outliers without making some assumption about the form of the calendar distribution (and which don’t fit well).

 Personally, I think you would be better fitting a standard uniform phase type model as that seems to effectively be what you have used as an argument to eliminate the two ^14^C samples with the oldest/youngest dates anyway.

**What is actually being modelled?**

 At some points in the paper you refer to your motivation being to perform an independent analysis of MBS 4. However, this doesn’t appear to be the actual model fitted as you have used samples from other units too.

 This is fine, but I am not sure that you can say that you are doing an independent analysis of MBS4 when most of the dates/samples that you are using for inference come from different Units entirely (4/16 from Unit 1; and 9/16 from Unit 2 (Pit 6)? In particular when the most recent date of your phase analysis (pre-outlier removal) seems primarily determined by your 1 ^st^ sample in Table 3 which comes from Unit 1:

 Unit 1, To-3/2, V19, #532 which has a ^14^C age of 14295 ^14^C yr BP (almost 500 ^14^C yrs more recent than all other samples)

 while the last is determined by SacA-12040 which comes from Unit 2, Pit 6 which has a ^14^C age of 15320 ^14^C yr BP.

**Failure to combine measurements on the same sample in model**

 It seems (from Fig 5 and Table 2) that the model does not combine measurements on the same sample (if this is what *idem* denotes) but treats them as though they are independent samples with potentially different calendar ages. For example, the model in Fig seems to consider GrA-38810 and SacA-11177 as though they are separate samples – whereas the Table suggests they are repeat measurements of the same sample and so must have the same calendar age. These repeated samples need to be combined (into a single sample) before being introduced into the model – not allowed to have two separate calendar ages.

**Suggestion that there are distinct clusters in Pit 6 and Toptalishche**

 On pg 12 you suggest there are distinct clusters in these two units. Is this based solely on Fig 5 (or from other information). Personally, I think it is hard to say confidently (based on Fig 5) that there are two distinct clusters. For example,  in Pit 6 Z2 there seem only 6 samples that have been analysed and they seem to all overlap in age. I would therefore remove this comment. Equally based on Fig 5 there isn’t much evidence that there are two distinct dating clusters in To3 based purely on 14C – there are only three samples analysed here. Or is this statement based on other evidence not presented here?

**Reproducibility/Availability of Code:**

 Currently there is no code or dataset attached to the paper (or at least in the version that I have access to). There is a suggestion of an SI but it’s not on the journal portal.  

**Specific Suggestions for Revisions/Edits:**

 To address the above comments, I would ideally like to see:

1. Model re-run ensuring *idem* measurements are modelled as having the same calendar age (if these relate to same actual object/sample). This is important.
2. Personally I would entirely ditch the KDE modelling component (as I am not sure what it actually means when included as part of a larger model) and just fit a traditional phase model that is much more easily explainable (both in terms of what the model is, and how it influences the analysis). At the least I would like to know that the results are robust to the KDE assumption. A more traditional parametric model may also allow you identify outliers in a more robust/rigorous manner.
3. Code and data (as e.g. csv file) should made available (ideally with a DOI via Zenodo). Data should not just be in a table in the manuscript. Even if there is an SI, I would suggest that storing it in a more accessible online repository is important for reproducibility.
4. Some relatively minor rewording about  (and further explanation) about why you have chosen to include some units but not others, and rephrasing to ensure you do not give the idea you have done an independent analysis of MBS4 (when much of the analysis seems influence by the other units). Currently it’s not entirely clear why you have chosen BMS 4 and Pit 6 (but not included, e.g., AMS dates from other pits)   

**Minor Points/Comments:**

1. Introduction - Please can you give specific/approximate calendar dates for the late pleniglacial period? This would aid readers who might not know this nomenclature/specific period to compare with other work more easily. This is especially critical as it only seems to be used as a term once – you later change to late Pleistocene and/or Paleolithic.
2. I found it slightly confusing when MBS 4 was first introduced (pg 3) as to what this was – I wasn’t sure if it was all four structures or just the 4 ^th^ one. I had to infer from the abstract it was just one of them. I would suggest that to clear this up, when you first introduce MBS in the paragraph before that you might name all four, i.e., *“A key site to this debate is Mezhyrich (Межиріч), situated in the Middle Dnieper Basin of Ukraine known for its exceptional preservation of four Mammoth Bone Structures (denoted in our manuscript as MBS 1, 2, 3 and 4 respectively) between 12–24 m2 in diameter (Figure 1).”* You might then need to be consistent with referring to MBSs (multiple structures) or a specific MBS. For example when referring to their first discovery (pg4) you could specify the initial excavations revealed MBS 1- 3; …
3. Pg 4 – Move up LGM abbreviation to 1 ^st^ usage of term last glacial maximum
4. Pg 8/9 – The three phases as described in the manuscript have overlapping calendar ages so I am confused why you say there are hiatuses between them – please can you explain?  
5. I do not understand the sub-analysis of phase timings on pg11 – are these obtained by rerunning the analysis again just on these samples? Or are they somehow obtained from the single initial model (i.e. you have start/end phases for all these other bits) 
6. Figure 5 – Suggest make clear from caption that this is the model after the *outliers* have been removed.
7. Conclusion – you have changed from using the term provenience to provenance. Is there a reason for this? Suggest you should be consistent.

Is the study design appropriate and does the work have academic merit?

Yes

Is the work clearly and accurately presented and does it cite the current literature?

Yes

If applicable, is the statistical analysis and its interpretation appropriate?

Partly

Are all the source data underlying the results available to ensure full reproducibility?

Partly

Are the conclusions drawn adequately supported by the results?

Partly

Are sufficient details of methods and analysis provided to allow replication by others?

Partly

Reviewer Expertise:

Radiocarbon; Statistics

I confirm that I have read this submission and believe that I have an appropriate level of expertise to confirm that it is of an acceptable scientific standard, however I have significant reservations, as outlined above.
